# Volatile Fatty Acids Production from Codigestion of Food Waste and Sewage Sludge Based on *β*-Cyclodextrins and Alkaline Treatments

**DOI:** 10.1155/2016/1698163

**Published:** 2016-12-14

**Authors:** Xue Yang, Xiang Liu, Si Chen, Guangmin Liu, Shuyan Wu, Chunli Wan

**Affiliations:** ^1^Postdoctoral Research Station of Civil Engineering, Tongji University, Siping Road, Shanghai 200092, China; ^2^State Key Laboratory of Pollution Control and Resources Reuse, College of Environmental Science and Engineering, Tongji University, Shanghai 200092, China; ^3^Department of Environmental Science and Engineering, Fudan University, Handan Road, Shanghai 200433, China; ^4^College of Materials Science and Chemical Engineering, Harbin Engineering University, Nantong Street, Harbin 150001, China; ^5^China Nerin Engineering Co., Ltd., Hainan Branch Company, Jinmao West Road, Hainan 570100, China

## Abstract

Volatile fatty acids (VFAs) are preferred valuable resources, which can be produced from anaerobic digestion process. This study presents a novel technology using *β*-cyclodextrins (*β*-CD) pretreatment integrated alkaline method to enhance VFAs production from codigestion of food waste and sewage sludge. Experiment results showed that optimized ratio of food waste to sewage sludge was 3 : 2 because it provided adequate organic substance and seed microorganisms. Based on this optimized ratio, the integrated treatment of alkaline pH 10 and *β*-CD addition (0.2 g/g TS) performed the best enhancement on VFAs production, and the maximum VFAs production was 8631.7 mg/L which was 6.13, 1.38, and 1.57 times higher than that of control, initial pH 10, and 0.2 g *β*-CD/g TS treatment, respectively. Furthermore, the hydrolysis rate of protein and polysaccharides was greatly improved in integration treatment, which was 1.18–3.45 times higher than that of other tests. Though the VFAs production and hydrolysis of polymeric organics were highly enhanced, the primary bacterial communities with different treatments did not show substantial differences.

## 1. Introduction

Food waste (FW) is becoming a serious concern for the developed countries due to its environmental impacts. About 6.0 × 10^7^ tons of FW were produced according to China Statistical Yearbook 2011, and the increasing rate of FW production was higher than 10% every year due to population growth and rising living standards. In addition, as the byproduct of wastewater treatment plants (WWTPs), 6.25 million tons of dry excess sludge has been produced in China in 2013 and is still showing a rapid increasing rate [1]. The excess sludge disposal has been considered as a main issue for the sustainable development of WWTPs because the cost of efficient WAS disposal is very high, accounting for approximately 40–60% operation fee of WWTPs [1, 2]. As well known, the main component of food waste and excess sludge is organic matter (e.g., proteins, polysaccharides, and lipid), and anaerobic digestion is preferred as an efficient pathway for treatment of these highly organic solids [3]. Efficient digestion of such organics can generate soluble organic products that assist in recovery of valuable resources [4]. Volatile fatty acids were valuable products as alternative carbons for nutrients removal, polyhydroxyalkanoates (PHA) production, and methane production processes [5–7].

Generally, anaerobic digestion process of particulate organic matter usually includes three stages: hydrolysis, acidification, and methanogenesis. Hydrolysis is known as the rate-limiting step. And only little of organic carbons can be biodegraded unless the particulate organic matter is significantly solubilized [8, 9]. Aiming at strengthening the performance of anaerobic digestion, some efforts have been developed, such as chemical, mechanical, biological, and thermal cofermentation treatments [10–14]. Alkaline treatment has been selected as a potential way for improving volatile fatty acids (VFAs) production and also affected the composition of hydrolysate, hence leading to distinct VFAs composition in the subsequent fermentation process [15]. However, though the VFAs production can be improved at pH 10, more than 60% of volatile suspended solids (VSS) could not be effectively degraded [15, 16]. Thus, it is necessary to study other methods to assist in alkaline pretreatment to further enhance VSS destruction and further harvest VFAs.

Some studies recently reported that surfactant can improve particulate organic matter dissolution and inhibit methanogenesis, which in turn benefitted VFAs production [17, 18]. Chemically synthesized surfactants (e.g., sodium dodecyl sulfate and sodium dodecyl benzenesulfonate) were firstly noted to improve the efficiency of anaerobic fermentation, but the residues present a potential risk to the ecosystem due to their biotoxicity [8, 11]. A typical *β*-cyclodextrin (*β*-CD) molecule has seven glucose monomers linked in a ring by *α*-1,4-glycosidic bonds [19], which could form host-guest complexes with hydrophobic molecules to enhance pollutant removal [20]. Compared with other chemical solubilizers, *β*-CD has low biologic toxicity and low potential to produce secondary pollutants [21]. Furthermore, our previous study approved that *β*-CD could enhance the hydrolysis rates of sewage sludge and inhibited activities of methanogens to maximize VFAs production [11].

The main objective of this study is to evaluate the feasibility of *β*-CD addition integrated into alkaline pretreatment for enhancing VFAs production from codigestion of food waste and sewage sludge. The mechanisms of integrated method for enhancing VFAs production were investigated by analyzing extracellular polymeric substances (EPS) and kinetic model of hydrolysis. Meanwhile, the bacterial communities of codigestion of FW and SS were studied to understand the codigestion of FW and SS based on the integrated treatment.

## 2. Methods and Materials

### 2.1. Food Waste and Sludge Inoculum

FW was collected daily from the cafeteria of a university located in Harbin (China). Grease was discarded through washing the FW for 3-4 times, and FW was crushed to particle size of 3–5 mm. SS was sampled from a municipal wastewater treatment plant in Harbin, China. The SS was firstly thickened by gravitational sedimentation for 24 h at 4°C and then screened to remove impurities. Finally, the FW and SS were stored at 4°C before use. The basic characteristics of FW and SS were listed in Table 1.

### 2.2. Batch Experiments for VFAs Production

Firstly, the optimized ratio of food waste (FW) to sewage sludge (SS) for VFAs production was determined. According to total solid (TS) listed in Table 1, the FW-to-SS ratios were 1 : 1, 2 : 1, 3 : 2, and 2 : 3, adjusting the final TS to 30 ± 0.5 g/L. Furthermore, the optimized dosage of *β*-CD for VFAs production was studied on the basis of the optimized ratio of FW to SS, and five parallel tests were conducted with *β*-CD addition in the range of 0–0.2 g/g TS. The pH was not adjusted in the above two tests. Then, to investigate the effects of alkaline pH, sole *β*-CD, and integrated treatments on VFAs production from FW and SS mixture, six parallel tests were carried out. The mixture without any pretreatments was adopted as control test (CK). Batch tests were conducted in 1 L serum bottles filled with 800 mL mixture of FW and SS. Based on the online pH monitor, the demanded pH was automatically pumped into the bottles by adding 4 M NaOH and 1 M HCl. The headspaces were flushed with nitrogen gas for 3–5 min to remove oxygen. Then the bottles were incubated in an air-bath shaker (120 rpm) at 35 ± 0.5°C for 8 days. All the experiments were carried out in triplicate.

### 2.3. Kinetics Modeling

 Pseudo-first-order (PFO) model was applied to understand the effects of sole or combined treatments on hydrolysis of polymeric substance during codigestion of FW and SS. The PFO model could be determined by the following equation:

(1) $$\begin{array}{c} \ln\left(\;C_{e}-C_{t}\;\right)=\ln C_{e}-kt, \end{array}$$

where *C*_*t*_ represents the concentration of protein/polysaccharides at time *t* (h), *C*_*e*_ represents the relative equilibrium capacity of protein/polysaccharides, and *k* (h^−1^) represents the rate constant of PFO model.

The normalized standard deviation (NSD, Δ*q* (%)) and average relative error (ARE (%)), calculated by the following equation, were employed to evaluate the error of PFO model [22, 23]:

(2) ARE%=100N−1∑i=1NCt,exp−Ct,calCt,expi2Δq%=1001N−1∑i=1NCt,exp−Ct,calCt,expi2,

where *C*_*t*,exp_ and *C*_*t*,cal_ represent the experimental and calculated values, respectively, at time *t* and *N* was the number of measurements made.

### 2.4. Bacterial Community Analysis

The total DNA (100 *μ*L) of samples collected from batch tests was extracted using Mo-Bio PowerSoil DNA Isolation Kit (Mo-Bio Laboratories, Inc., USA) according to the manufacturer's protocol. The genomic DNA was evaluated by electrophoresis in 1% agarose gels.

The PCR procedures were referred to in Wan et al. [24] and the mixture (50 *μ*L) consisted of the following: 5 *μ*L of 10x PCR buffer, 0.5 *μ*L of dNTP (10 mM each), 10 ng of genomic DNA, 1 *μ*L of Bar-PCR primer F (50 *μ*M), 1 *μ*L of primer R (50 *μ*M), 0.5 *μ*L of Platinum Taq (5 U/*μ*L), and sterile ddH_2_O to a final volume of 50 *μ*L. For the following high-throughput sequencing, the PCR primers were Bar-PCR primer F (CGTATCGCCTCCCTCGCGCCATCAG + BACODE + AGRGTTYGATYMTGGCTCAG) and primer R (CTATGCGCCTTGCCAGCCCGCTCAG + ACCGCGGCKGCTGGC). The PCR conditions were as follows: 94°C for 10 min; 5 cycles consisting of 94°C for 20 s, 45°C for 20 s, and 65°C for 60 s; 20 cycles consisting of 94°C for 20 s, 60°C for 20 s, and 72°C for 20 s; and a final step of 10 min at 72°C. The PCR products were evaluated using 1% agarose gels. The PCR products were further purified using a DNA gel extraction kit (Sangon Biotech Co., Ltd., Shanghai, China). Then, the extracted PCR products were quantified using Qubit 2.0 kit. Finally, all the PCR products were doubly diluted by sterile ddH_2_O.

The ROCHE Emulsion-PCR technology was adopted to prepare single-molecule PCR product. Emulsion-PCR (e-PCR) mixture could integrate PCR aqueous phase with oil phase. Each mixed droplet contained one molecule of DNA, magnetic bead, and PCR reaction mixture. Therefore, PCR bias can be effectively reduced due to the efficient amplification of one molecule of bacterial DNA. Finally, the genomic samples were taken for high-throughput sequenced by Ion Torrent PGM (Life Technologies, USA).

### 2.5. Analytical Methods

Collected samples were firstly centrifuged at 10,000 rpm for 10 min; then supernatant samples were filtered by 0.45 *μ*m membrane filters and finally filtrated samples were stored at 4°C prior to analysis. The filtrate was immediately used to analyze VFAs, polysaccharides, and proteins. The measurements of VFAs, SCOD, TCOD, polysaccharides, and proteins were the same as the methods mentioned in previous publications [8, 25, 26]. The VFAs were regarded as the sum of acetic (HAc), propionic (HPr),* n*-butyric (*n*-HBu),* iso*-butyric (*iso*-HBu), n-valeric (n-HVa), and isovaleric acids (iso-HVa) [11].

## 3. Results and Discussion

### 3.1. Performances of VFAs Production from Codigestion of FW and SS

#### 3.1.1. Optimized FW-to-SS Ratio for VFAs Production

**Figure 1:**
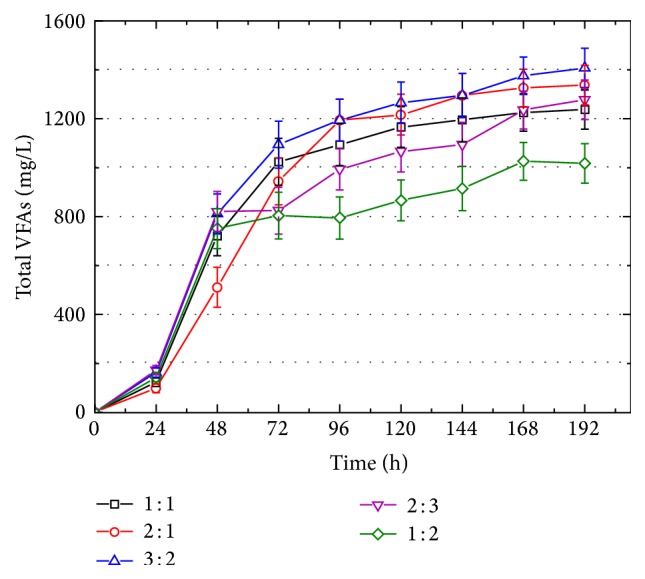
Performances of VFAs production from codigestion of FW and SS at different ratios.

In order to determine the optimized ratio of FW and SS for VFAs production, five ratios of FW and SS (1 : 1, 2 : 1, 3 : 2, 2 : 3, and 1 : 2) (TS) were conducted (Figure 1). The highest VFAs (1401.4 mg/L) at the end of cofermentation were obtained at the ratio of 3 : 2 (TS), followed by 1337.4 mg/L of VFAs at ratio of 2 : 1. Then, the VFAs production at ratios of 2 : 3 and 1 : 1 was almost the same (1277.4 versus 1237.9 mg/L), which were both higher than the test at ratio of 1 : 2 (1014.7 mg/L). Although the TS in all tests was around 30 g/L, more FW addition brought more VFAs. The food waste contained more easily biodegradable substances than sewage sludge, and sludge provided microorganisms to degrade these polymeric organics. Thus, more food waste addition brought VFAs production. And, higher VFAs yield at ratio of 3 : 2 than 2 : 1 suggests that adequate sludge was also necessary for VFAs production.

#### 3.1.2. Effects of Different *β*-CD Dosages on VFAs Production

**Figure 2:**
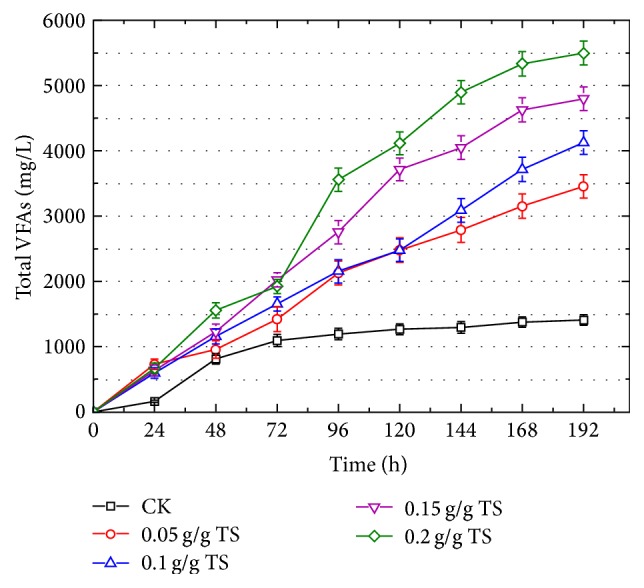
Effects of *β*-CD dosage (including 0.05, 0.1, 0.15, and 0.2 g *β*-CD/g total solid (TS)) on total VFAs production from mixture of FW and SS at ratio of 3 : 2. The test without *β*-CD addition was set as control test (CK).

As shown in Figure 2, it was clear that the *β*-CD addition improved VFAs production with all ranges of *β*-CD dosages. The minimal *β*-CD addition (0.05 g/g TS) obtained 3456.1 mg/L of VFAs, which was 2.46 times than that of control test. With the increase in *β*-CD addition, more VFAs were accumulated at the end of fermentation, and 4126.9 and 4796.9 mg/L of VFAs were, respectively, obtained with 0.1 g/g TS and 0.15 g/g TS *β*-CD addition. The maximal VFAs production was achieved with 0.2 g/g TS addition, which was 3.9 times higher than that obtained in control test. It was interesting that VFAs production did not show substantial differences with different *β*-CD addition during the first 72 h but indicated obvious increasing during the last days. *β*-CD is comprised of 7 *α*-D-glucopyranoside units and has hydrophobic groups inside and hydrophilic group outside, and the cavity of *β*-CD is 6.0–6.5 × 7.9 Å. It can enhance solubility of hydrophobic compounds with smaller molecules than the cavity of *β*-CD, which in turn solubilize the particulate organic substance in this study [11]. Due to the adhesive characters of EPS, the sewage sludge or food waste has enormous aggregated molecules. Along with microbial metabolism, the enormous extracellular aggregates were broken into pieces, and then the polymers deserved solubilization by *β*-CD. Thus, if other more aggressive treatments could be synchronously adopted with *β*-CD addition, the acclimation phase might be shortened.

#### 3.1.3. Effects of Different Treatments on VFAs Production

**Figure 3:**
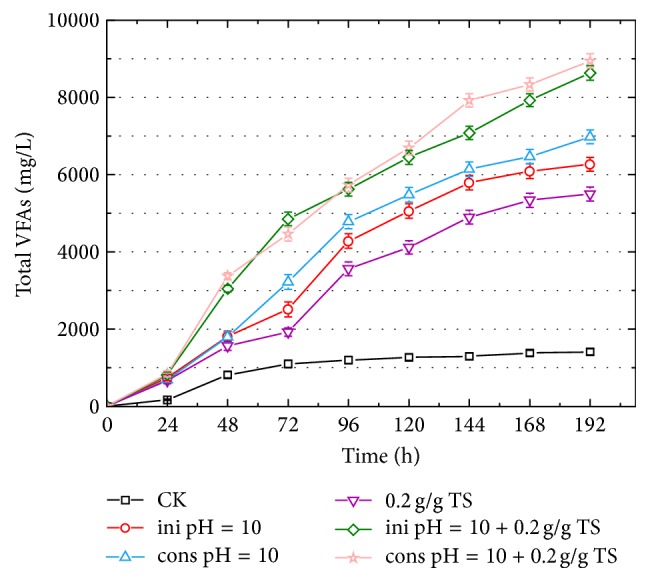
Effects of alkaline pH 10, *β*-CD addition, and combined treatment on VFAs production from codigestion of FW and SS (3 : 2). The pH control was divided into initial pH 10 adjustment (ini pH = 10) and constant pH 10 adjustment (cons pH = 10). The *β*-CD dosage was 0.2 g/g total solid (TS).

Previous studies have reported that VFAs production from sewage sludge could be obviously enhanced either by adopting an initial pH 10 or by controlling pH at constant 10 [15]. This study compared the effects of these two alkaline conditions on VFAs production from codigestion of FW and SS. As shown in Figure 3, the VFAs production at initial pH 10 was 6262.3 mg/L, which was 4.45 times higher than that obtained in control test, indicating the potential application of initial pH 10 for VFAs production from FW and SS fermentation. And the VFAs production at the end of fermentation was 6971.3 mg/L, which was 1.11 times higher than that under initial pH 10. The analogous results were obtained at combined treatments of initial/constant pH 10 with 0.2 g *β*-CD/g TS, which, respectively, produced 8631.7 mg/L (ini pH 10 + 0.2 g/g TS) and 8943.1 mg/L (cons pH 10 + 0.2 g/g TS). Despite higher VFAs production with constant pH 10, constant alkaline chemical addition was neither economic nor convenient for organic wastes disposal. Furthermore, it was obvious that VFAs production was promoted by sole *β*-CD addition, which was 3.9 times higher than that of control test. However, the VFAs production by sole *β*-CD addition was lower than that obtained by sole alkaline pH 10, which might be due to the gentler solubilization of organic substances by *β*-CD than by alkaline pH. But, considering the corrosion of pipes or equipment by external regents, *β*-CD addition was more promising than alkaline treatments.

#### 3.1.4. Composition of VFAs with Different Treatments

**Figure 4:**
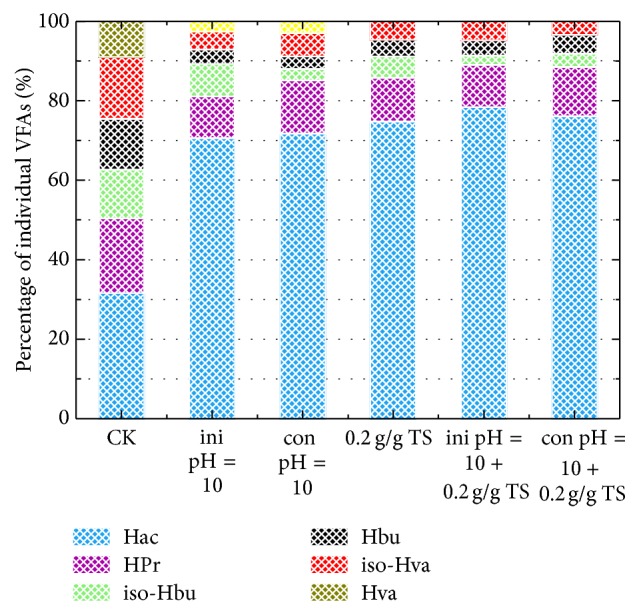
Composition of VFAs from codigestion of FW and SS (3 : 2) with different treatments at 8 d. The pH control was divided into initial pH 10 adjustment (ini pH = 10) and constant pH 10 adjustment (cons pH = 10). The *β*-CD dosage was 0.2 g/g total solid (TS). The mixture of FW and SS without any treatment was set as control test (CK).

The composition of VFAs would influence the further application of fermentation liquid, such as external carbon source for nutrient removal [27]. It was observed that the composition of VFAs was significantly affected by alkaline pH and *β*-CD addition. For all the tests, HAc and HPr were the two highest individual VFAs with a total percentage of 81.1%–89.0%, which was 1.61–1.77 times higher than that of control test (Figure 4). The order of percentage of individual VFAs was HAc > HPr > Hbu > Hva. The possible reasons were that HAc and HPr were directly from the fermentation of organic polymers [26], and higher molecular weight VFAs such as Hva or Hbu were easily biodegraded to form HAc in anaerobic fermentation system [15, 27].

### 3.2. Mechanisms of VFAs Production Based on Combined Treatments

#### 3.2.1. Kinetic Modeling of Hydrolysis of Polymeric Substances

**Figure 5:**
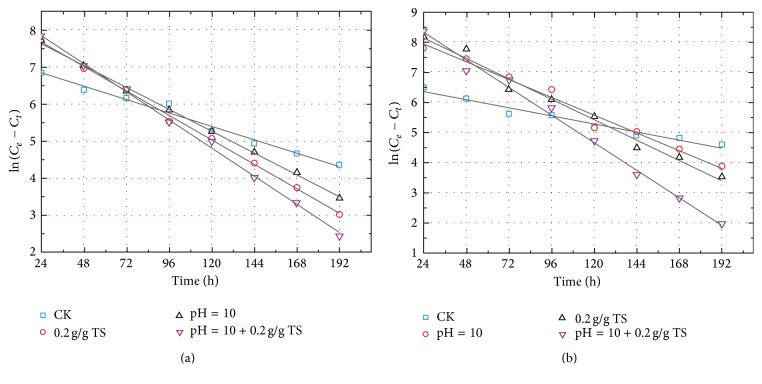
Kinetic modeling of hydrolysis of FW and SS based on different treatments. (a) and (b), respectively, represent kinetic analysis of protein and polysaccharides. The pH control was initial pH 10 adjustment. The *β*-CD dosage was 0.2 g/g total solid (TS). The mixture of FW and SS without any treatment was set as control test (CK).

As the VFAs production by constant pH 10 did not indicate much more advantages than by initial pH 10, thus in the mechanisms of VFAs production was only investigated in initial pH 10 tests. The levels of soluble protein and polysaccharides could be taken as an index to evaluate the efficiency of hydrolysis. Thus, the released proteins/polysaccharides were fitted using PFO model, evaluating the hydrolysis of polymeric substances during codigestion of FW and SS. Figures 5(a) and 5(b) indicate the hydrolysis kinetics of FW and SS based on sole and combined treatments. It was obvious that good agreement was achieved between calculated and experimental results, with strong correlation coefficients (*R* ^2^: 0.962–0.999), low ARE (%) (0.024–0.900 < 1), and Δ*q* (%) (1.553–9.490 < 10) (Table 2), suggesting that the hydrolysis of protein/polysaccharides from the mixture (FW and SS) obeyed the PFO model. In terms of sludge, both PFO and first-order models have been successfully used to fit the hydrolysis of polymeric substances [15, 28]. The highest hydrolysis rate constants of protein and polysaccharides were obtained in combined tests, 2.13 (protein) and 3.45 (polysaccharides) times higher than control test. The sole pH 10.0 treatment took more obvious enhancement on hydrolysis of protein than that of polysaccharides, which was similar to the results of hydrolysis of sewage sludge [15]. Contrary to the protein, the polysaccharides deserved more obvious hydrolysis based on the sole *β*-CD treatment (0.2 g/g TS), which was mainly due to the analogous molecular structure of *β*-CD to polysaccharides. Furthermore, both alkaline and *β*-CD treatments boosted hydrolysis of protein and polysaccharides compared to control test.

#### 3.2.2. Evolution of Bacterial Communities

Detailed evolution of bacterial communities was investigated to further find out the underlying mechanisms of the difference between the effects of alkaline pH and *β*-CD addition on anaerobic codigestion of FW and SS. After Chimer analysis, 33561, 37823, and 30222 reads were generated for further analysis. These high quality reads were assigned to different taxa levels using the RDP classifier. A total of 25 phyla were identified, and* Proteobacteria*,* Bacteroidetes*,* Actinobacteria*, and* Firmicutes* were the predominant phyla in bacterial community.

The top 10 abundant genera (Table 3) in each sample were selected for a detailed understanding of the evolution of microbial community structure as suggested previously [29].* Parabacteroides* obviously increased from 0.15% in initial sludge to 17.37%–27.11% in all three tests and in turn became the predominant member in all three tests. It was more probably related to its function as a saccharolytic bacterium that produces acetate and succinate as primary fermentation end products [30].* Proteiniborus* was the second predominant member in all three tests at the end of codigestion, which is usually recognized as protein-specific utilizing bacteria, and the fermentation products mainly include ethanol, acetic acid, hydrogen, carbon dioxide, and a trace amount of propionic acid [31].* Petrimonas* increased with the fermentation time and showed highest abundance in integrated treatment test. Members belonging to this genus are mesophilic, strictly anaerobic, and fermentative bacteria, and acetic acid was the major end product [32].* Sporacetigenium* was detected as main member in *β*-CD addition test but not abundant in alkaline pH and integrated experiments, which was mainly because it plays an important role in producing acetate and butyrate from glucose but cannot survive with strong alkaline pHs [33].* Butyrivibrio* and* Eubacterium* were not main members in bacterial samples at initial and 3 d but accumulated at the end of fermentation in all three tests. They belong to Clostridia, and the main members played an important role in butyric acid type fermentation [34]. This accumulation of* Butyrivibrio* and* Eubacterium *might be due to the acclimation of nutrient conditions. The other main coexisting bacteria included* Propionibacterium*,* Terrimonas*,* Moorella*, and* Nitrospira*. They survived in the whole codigestion process but did not become the primary ones in bacterial samples. The function of these coexisting bacteria was mainly VFAs production with propionic acid and acetic acid as fermentation products.

## 4. Conclusions

The feasibility of short-term codigestion for enhancing VFAs production from FW and SS with a novel proposed method, *β*-CD addition integrated into alkaline pH 10, has been investigated in this work. The main conclusions are as follows. (1) Optimized ratio of FW to SS was 3 : 2 because the system could provide adequate organic substance and seed microorganisms. (2) Positive synergies on anaerobic codigestion were obtained. The maximum VFAs production was 8631.7 mg/L at the end of fermentation, when FW and SS were pretreated by initial pH 10 and *β*-CD dosage of 0.2 g/g TSS. (3) All the treatment methods (pH 10, *β*-CD addition, and integrated methods) could enhance the release EPS to suspension, and the highest hydrolysis rates for protein and polysaccharides were 0.032 and 0.038 (based on integrated method). (4) Primary bacterial communities based on each treatment did not show substantial difference, which are comprised of* Parabacteroides*,* Proteiniborus*,* Petrimonas*, and* Sporacetigenium*.

## Figures and Tables

**Table 1:** The characteristics of food waste and feed sludge for anaerobic codigestion.

Characters	Food waste	Sewage sludge
Total solids (g/L)	31.23 ± 0.43	29.72 ± 0.47
Volatile solids (g/L)	30.34 ± 0.38	23.67 ± 0.31
VFAs (mg/L)	17.55 ± 3.81	64.19 ± 7.61

**Table 2:** Kinetic parameters for hydrolysis based on different treatments.

	Hydrolysis of protein				Hydrolysis of polysaccharides			
	CK	pH = 10	0.2 g/g TS	pH = 10 + 0.2 g/g TS	CK	pH = 10	0.2 g/g TS	pH = 10 + 0.2 g/g TS
*k*_1_ (h^−1^)	0.015	0.027	0.025	0.032	0.011	0.024	0.028	0.038
*C*_*e*_	1421	3855	3058	4753	939	2857	3836	4664
*R* ^2^	0.991	0.998	0.999	0.998	0.962	0.977	0.981	0.991
Δ*q* (%)	2.314	1.758	2.212	1.553	8.973	9.490	8.461	9.097
ARE (%)	0.049	0.031	0.049	0.024	0.805	0.900	0.715	0.827

**Table 3:** Top 10 bacterial genera from codigestion of FW and SS (3 : 2) with different treatments at 3 d and 8 d. Unclassified sequences were removed prior to the data analysis (%, relative abundance).

Genera	Initial	pH 10/3 d	*β*-CD/3 d	Integrated/3 d	pH 10/8 d	*β*-CD/8 d	Integrated/8 d
*Parabacteroides*	0.15	19.60	17.37	23.98	24.32	21.29	27.11
*Proteiniborus*	1.02	15.66	10.83	17.31	17.46	13.82	18.05
*Petrimonas*	0.60	4.67	5.9	7.07	6.43	7.55	9.95
*Sporacetigenium*	2.18	0.25	7.98	0.41	0.15	7.37	0.32
*Butyrivibrio*	0.13	1.08	1.65	1.00	4.37	5.94	3.28
*Eubacterium*	0.12	1.12	2.02	0.84	2.21	2.65	2.07
*Propionibacterium*	0.33	0.67	0.90	0.43	0.41	0.55	0.73
*Terrimonas*	0.41	0.84	0.72	0.32	0.45	0.35	0.81
*Moorella*	0.31	0.59	0.61	0.88	0.76	0.74	1.33
*Nitrospira*	0.12	0.44	0.32	0.33	0.61	0.67	0.35
